# Meningeal and Pulmonary Tuberculosis in an Adult

**Published:** 1877-04

**Authors:** J. H. Wm. Meyer

**Affiliations:** House Physician to Cook County Hospital


### MENINGEAL AND PULMONARY TUBERCULOSIS IN AN ADULT.

By DR. J. H. WM. MEYER.

[House Physician to Cook County Hospital.]

Ellebert Leganger, a Norwegian silversmith, was admitted Dec. 16, 1876, suffering from tuberculosis and chronic pleurisy. While delirious, patient destroyed the record of his case.

An examination was made six hours after death, which occurred, Feb. 17, 1877. The right pleural cavity was found to contain about 3 quarts of light yellow fluid; the pleura was much thickened, and presented several strong bands of adhesion; the right lung was compressed to about one-third its normal size, and thickly studded, from apex to base, with miliary tubercles; there was a small cavity in its apex.

On the left side, the two layers of pleura were adherent throughout; the left lung also was completely filled with miliary tubercles. The dura mater, arachnoid and pia mater were greatly congested; arachnoidean cavity contained a very small amount of liquid. There was also a slight serous effusion into the ventricles.

Found a deposit of semi-transparent, gray tubercles along the course of the vessels, especially on the convex surface of the hemispheres; there was a similar deposit, slight in amount, at the base; vascular turgescence of the cerebral tissues, shown by the increased size of the puncta. A specimen of the lung tissue was submitted to Dr. Danforth for microscopic examination.

*Microscopy.*—The most prominent feature revealed by the microscope was the hypertrophied condition of the intervesicular connective tissue, and the general infiltration in the small, shrivelled, irregular cells. In many instances, these cells were filled with pigment, and these pigmented cells were arranged in dense groups, or else stretched out into continuous lines or columns. In some places, they formed a continuous ring, surrounding the air vesicle, and infiltrating the intervesicular connective tissues. The air vesicles were pretty generally deprived of their epithelium, but they were stuffed with a luxuriant crop of rather small, irregular, imperfectly formed cells.

The tunica adventitia of the blood vessels was thickened, and the arteriolæ were abnormally tortuous. I. N. D.

